# Effects of worn and new footwear on plantar pressure in people with gout

**DOI:** 10.1186/s12891-021-04370-x

**Published:** 2021-05-24

**Authors:** Mike Frecklington, Nicola Dalbeth, Peter McNair, Alain Vandal, Peter Gow, Keith Rome

**Affiliations:** 1 Health and Rehabilitation Research Institute, AUT University, Private Bag 92006, Auckland, 1142 New Zealand; 2 The University of Auckland, Private Bag 92019, Auckland, 1142 New Zealand; 3 Auckland District Health Board, P.O. Box 92189, Auckland, New Zealand; 4 Department of Biostatistics & Epidemiology, Auckland University of Technology, Private Bag 92006, Auckland, 1142 New Zealand; 5 Research & Evaluation Office, Ko Awatea, Counties Manukau Health, Private Bag 93311, Auckland, 1640 New Zealand; 6 Counties Manukau District Health Board, Private Bag 94052, Auckland, 2240 New Zealand

**Keywords:** Gout, Footwear, Plantar pressure

## Abstract

**Background:**

In clinical trials, good quality athletic shoes offer short-term improvements (two-months) in foot pain and disability in people with gout, but these improvements are not sustained over time. This may be due to wear and subsequent changes to the structural integrity of the shoe. The aim of this study was to examine the effects of wear on plantar pressures and footwear characteristics in shoes over six-months in people with gout.

**Methods:**

Forty people with gout participated in a cross-sectional repeated measures study. Participants wore a pair of commercially available athletic footwear for six-months. Participants then attended a study visit where the worn footwear was compared with a new pair of the same model and size of footwear. Wear characteristics (upper, midsole, outsole) and plantar pressure were measured in the two footwear conditions. Wear characteristics were analysed using paired t-tests and Fisher’s exact tests. Plantar pressure data were analysed using linear mixed models.

**Results:**

Increases in medial midsole (*P* < 0.001), lateral midsole (*P* < 0.001) and heel midsole (*P* < 0.001) hardness were observed in the worn shoes. Normal upper wear patterns (*P* < 0.001) and outsole wear patterns (*P* < 0.001) were observed in most of the worn shoes. No differences in peak plantar pressures (*P* < 0.007) were observed between the two footwear conditions. Reduced pressure time integrals at the first metatarsophalangeal joint (*P* < 0.001), second metatarsophalangeal joint (*P* < 0.001) and hallux (*P* = 0.003) were seen in the worn shoes.

**Conclusions:**

The study found signs of wear were observed at the upper, midsole and outsole in the worn footwear after six-months. These changes to the structural properties of the footwear may affect forefoot loading patterns in people with gout.

## Background

People with gout experience high levels of foot pain, impairment and disability [1]. A large proportion of people with gout wear inappropriate footwear that is worn, lacks cushioning and support, which is associated with pain and disability [2]. Footwear containing cushioning and support has been found to improve foot pain, impairment and disability over an eight-week period in people with gout, with no changes observed in those wearing footwear lacking these features [3]. These improvements in patient reported outcomes may be attributed to the characteristics of the footwear influencing loading patterns [4].

People with gout have altered loading patterns during walking when shod [4, 5] and barefoot [6]. When wearing their own footwear, people with gout display reduced peak plantar pressures and pressure time integrals at the hallux and increased pressure time integrals at the midfoot compared to controls [5]. In people with gout, footwear characteristics that included dual density midsole, heel and forefoot cushioning and a rocker-sole reduced peak plantar pressures and pressure time integrals at the third and fifth metatarsals and heel, and increased pressure time integrals at the midfoot, compared to the participant’s own footwear [4]. Postulated factors contributing to first metatarsophalangeal joint involvement in gout include lower temperatures, trauma, biomechanical loading and the co-existence of osteoarthritis [7]. Therefore, these changes in pressure may reflect a pain avoidance strategy where people with gout offload painful regions of the feet [5].

Although previous work has found short-term benefits in pain and disability with footwear [3], a recent clinical trial found that these were not sustained over a six-month period [8]. In this trial, participants with gout were randomised to receive either podiatric care and gout education (control group) or podiatric care, gout education and commercially available athletic footwear (footwear intervention group). Between-group improvements in pain, impairment and disability were seen at two-months with the footwear intervention, however, these were not observed at six-months. This may be due to wear and subsequent changes to the structural integrity of the footwear resulting in changes in plantar loading. The aim of this study was to examine the effects of six-months of wear on plantar pressure and footwear characteristics in people with gout.

## Methods

### Participants

A cross-sectional repeated measures study was undertaken. Participants with gout were recruited through newspaper advertising and from rheumatology clinics in Auckland, New Zealand. Inclusion criteria were: gout according to the 1977 preliminary American Rheumatism Association classification criteria [9] and over 20 years of age. Exclusion criteria were: history of other inflammatory arthritis, history of neuromuscular disease, experiencing a gout flare at time of assessment, medication for foot pain in the past month, history of foot or ankle surgery, or unable to walk 10 m unaided. Participants were fitted with a new pair ASICS Cardio Zip 3 footwear. The appropriate footwear size was determined using a Brannock device and then fitted by a podiatrist [MF]. All participants wore footwear for six-months, with self-reported diaries used to record the number of hours the footwear was worn per week. These diaries have been used in previous gout studies [3]. Participants then returned for a study visit in which they were tested with the worn shoes and a new pair of the same model of shoes. Ethical approval was obtained from the Auckland University of Technology Ethic Committee (AUTEC 14/233). All participants provided written consent.

### Interventions

Two footwear conditions were evaluated; (1) a pair of commercially available athletic footwear that had been worn for 6 months (worn footwear) and; (2) a new pair of the same model of footwear (new footwear). This footwear was chosen based on the findings of a previous feasibility study [3], and its characteristics including heel/forefoot cushioning, dual density midsole, wide-fitting option and a zip for ease of fit. The footwear tested was the same size and model for both footwear conditions. For participants with existing foot orthoses, the sock-liner of the footwear was removed and replaced by the orthoses for testing.

### Procedure

All assessments were undertaken by a single researcher [MF]. Assessments took place six-months after the initial footwear fitting, except for the assessment of the new footwear that took place prior. Worn and new footwear were assessed using the Footwear Assessment Tool [10]. Heel height and forefoot height (mm) were both measured using digital callipers. Heel height was the average of heights measured at the medial and lateral heel. Forefoot height was the average of heights measured at the level of the first and fifth metatarsals. Heel counter stiffness, midfoot sagittal stability and midfoot torsional stability were categorised as either minimal (> 45°), moderate (< 45°) or rigid (< 10°). Heel counter stiffness was determined by applying a force to the posterior aspect of the heel counter. Midfoot sagittal stability was determined by bending the shoe at the midfoot in sagittal plane. Midfoot frontal stability was determined by twisting the shoe in the frontal plane at the midfoot. Midsole hardness was measured using a Shore A durometer. Lateral midsole hardness was measured at the lateral midsole at the level of the heel. Medial midsole hardness was measured at the medial midsole at the level of the heel. Heel sole hardness was measured at the inferior aspect of the heel inside the shoe. Upper wear was categorised as either neutral, medial tilt (greater than 10°), or lateral tilt (greater than 10°). Midsole wear was categorised as either neutral, medial midsole compression or lateral midsole compression. Tread pattern was categorised as either no wear, partly worn or fully worn. Outsole wear was categories as either neutral (wear from lateral heel to medial forefoot), medial (greater medial wear at the heel/forefoot), or lateral (greater lateral wear at the heel/forefoot).

The following clinical characteristics were collected on the day of the assessment visit; age, gender, ethnicity, body mass index (BMI), foot posture index, history of diabetes, cardiovascular disease and peripheral vascular disease, latest serum urate, disease duration, number of gout flares in previous 3 months, presence of subcutaneous tophus, presence of foot subcutaneous tophus and current pharmacological management.

Prior to randomisation of the footwear conditions to be tested (new and worn footwear), participants were instructed to walk across the GAITRite® walkway (CIR Systems, Inc., New Jersey, US) at a self-selected speed to determine the participant’s normal walking speed [11]. GAITRite is a 700 cm × 90 cm electronic walkway with an active sensor area of 609.6 cm long and 60.96 cm wide. The active area contains sensor pads (23,040 pressure activated sensors), with a spatial resolution of 1.27 cm and a sampling rate of 120 Hz. The average of three trials was used to determine the participant’s normal walking speed [6].

Testing order of the footwear conditions was randomised using unstratified block randomisation. The primary outcome was plantar pressure (peak plantar pressure and pressure time integrals), measured using the F-Scan® Mobile system (Tekscan Inc., South Boston, MA, USA). Each insole contains 954 sensors (3.9 sensors per 1cm^2^) and was placed on top of the sockliner. The system was calibrated prior to data acquisition [12], with data obtained using the five-stride protocol [4, 5]. Three trials were completed in both pairs of footwear with seated breaks between trials. Walking speed was monitored during each trial using the GAITRite walkway. Following each trial, if the walking speed was 5% outside of the average self-selected speed determined prior, participants were asked to repeat the trial [13]. The FScan® software package (Tekscan Inc., Version 5.24) was used to analyse the plantar pressure data (five steps per foot side). The foot was manually masked into 7 regions (heel, midfoot, first metatarsal (1MTP), second metatarsal (2MTP), lesser metatarsals (345MTP), hallux and lesser digits), mean peak plantar pressure (kPa) and pressure time integrals (kPa*sec) were calculated. These measures have been found to be reliable in the gout population [5].

### Sample size calculation

The sample size assessment is based on a previous plantar pressure study of gout and footwear [4]. In this study, the participant’s own shoes and the intervention footwear were worn on the same visit and plantar pressure measurements taken under both conditions. Plantar pressure was remeasured at 8 weeks with the intervention footwear that had been in use during this period (unpublished data). The standard deviation of the differences was 152 kPa. A sample size of 40 allows the detection of a difference of 69 kPa (effect size 0.45) between new and worn intervention footwear with 80% power at a significance level of 5% using a paired t-test. The use of a linear mixed model on repeated measures makes this power assessment conservative.

### Statistical analysis

Linear mixed models were used to determine differences between plantar pressure and walking velocity and the two footwear conditions: worn footwear and new footwear. The two footwear conditions were entered as fixed effects, with the paired-foot data (left side and right side) and variables measured entered as random effects [14]. These models account for repeated measures taken from the left and right feet. Walking velocity was not paired for foot side. Paired *t*-tests and Fisher’s exact tests were used to test for statistical differences in the categories of the footwear assessment form. No adjustments for covariates were made as participants acted as their own control. Significance at the 0.05 level was declared accounting for a Bonferroni correction based on the 7 plantar pressure outcomes (*P* < 0.007) and 12 footwear outcomes (*P* < 0.004). All tests were carried out against two-sided alternatives. Data were analysed using SAS/STAT™ software version 9.4.

## Results

### Study participants

A total of 40 people with gout participated in the study. The majority of participants were European with a mean (SD) age of 67 (13) years and disease duration of 13 (12) years (Table 1). The mean (SD) BMI was 30.5 (6.5) kg/m^2^. Over the six-months, footwear diaries were completed in 2 month intervals, with 21 participants returning all completed diaries, 13 participants returning at least 1 completed diary and 6 participants did not complete any footwear diaries. Due to missing data, hours worn were not included in the models. For those participants who returned at least one diary, the mean (SD) duration of wearing the footwear was 20 (15) hours per week. One participant withdrew during testing due to discomfort meaning there was missing data for plantar pressure in the worn footwear.

**Table 1:** Demographic and clinical characteristics

Variable	Summary
Sex (male), n (%)	35 (88%)
Age (years), mean (SD)	67 (13)
BMI (kg/m^2^), mean (SD)	30.5 (6.5)
Foot posture index, mean (SD)	4 (4)
Ethnicity, n (%)	
European	30 (75%)
Pacific peoples	4 (10%)
Māori	3 (8%)
Asian	3 (8%)
Gout history	
Disease duration (years), mean (SD)	13 (12)
Self-reported flares in previous 3 months, mean (SD)	0.4 (0.8)
Foot tophus, n (%)	12 (30%)
Any tophus, *n* (%)	15 (35%)
Serum urate, mmol/L, mean (SD)	0.34 (0.11)
Medications, n (%)	
Urate lowering therapy	25 (63%)
Colchicine	8 (20%)
Prednisone	8 (20%)
NSAID	14 (35%)
Diuretic	6 (15%)
Medical history, n (%)	
Hypertension	21 (53%)
Cardiovascular disease	12 (30%)
Diabetes	4 (10%)
Peripheral vascular disease	3 (8%)

*BMI* Body mass index, *NSAID* Non-steroidal anti-inflammatory drug

### Footwear characteristics

Reductions in heel height by 3% (*P* < 0.001), forefoot height by 5% (*P* < 0.001), heel counter stiffness (*P* < 0.001), midfoot sagittal stiffness (*P* < 0.001) and midfoot frontal stiffness (*P* = 0.001) were observed in the worn footwear (Table 2). Increases in medial midsole hardness by 4% (*P* < 0.001), lateral midsole hardness by 2% (*P* < 0.001) and heel midsole hardness by 5% (P < 0.001) were observed in the worn footwear. Signs of outsole wear was evident in the worn footwear, with the majority displaying normal upper (*P* < 0.001), midsole (*P* = 0.005) and outsole (*P* < 0.001) wear patterns.

**Table 2:** Footwear characteristics

Characteristic^a^	New Shoe	Worn Shoe	*P*	% change
Heel height (mm), mean (SD)	37.3 (0.9)	36.2 (1.2)	**< 0.001**	3%
Forefoot height (mm), mean (SD)	20.6 (0.5)	19.4 (0.6)	**< 0.001**	5%
Heel counter stiffness, n (%)			**< 0.001**	NA
Minimal (> 45°)	0 (0%)	1 (3%)		
Moderate (< 45°)	0 (0%)	12 (30%)		
Rigid (< 10°)	40 (100%)	27 (68%)		
Midfoot sagittal stiffness, n (%)			**< 0.001**	NA
Minimal (> 45°)	0 (0%)	1 (3%)		
Moderate (< 45°)	0 (0%)	18 (45%)		
Rigid (< 10°)	40 (100%)	21 (53%)		
Midfoot frontal stiffness, (n%)			**0.001**	NA
Minimal (> 45°)	0 (0%)	1 (3%)		
Moderate (< 45°)	0 (0%)	9 (23%)		
Rigid (< 10°)	40 (100%)	30 (75%)		
Lateral midsole hardness (Shore A), mean SD)	57.0 (0)	58.3 (0.9)	**< 0.001**	2%
Medial midsole hardness (Shore A), mean (SD)	54.0 (0)	56.2 (1.2)	**< 0.001**	4%
Heel midsole hardness (Shore A), mean (SD)	56.0 (0)	58.8 (1.3)	**< 0.001**	5%
Upper wear, n (%)			**< 0.001**	
None	40 (100%)	0 (0%)		
Medial tilt	0 (0%)	17 (43%)		NA
Neutral	0 (0%)	23 (58%)		
Lateral tilt	0 (0%)	0 (0%)		
Midsole wear, n (%)			0.005	NA
None	40 (100%)	0 (0%)		
Medial	0 (0%)	8 (20%)		
Neutral	0 (0%)	32 (80%)		
Lateral	0 (0%)	0 (0%)		
Tread, n (%)			**< 0.001**	NA
Not worn	40 (100%)	0 (0%)		
Partly worn	0 (0%)	40 (100%)		
Fully worn	0 (0%)	(0%)		
Outsole wear, n (%)			**< 0.001**	NA
None	40 (100%)	0 (0%)		
Medial	0 (0%)	0 (0%)		
Normal	0 (0%)	40 (100%)		
Lateral	0 (0%)	0 (0%)		

^a^data presented for left shoe only

### Plantar pressure measurements

No significant differences in peak plantar pressure were observed across the seven regions of the foot (Table 3). However, reductions in pressure time integrals of 9% at the 1MTP (*P* < 0.001), 6% at the 2MTP (*P* < 0.001) and 7% at the hallux (*P* = 0.003) were observed in the worn footwear compared to the new footwear (Table 4). No significant differences in pressure time integrals were observed across the other masked regions (*P* > 0.007).

**Table 3:** Peak plantar pressure (kPa)

Parameter	Condition	Mean (SD)	Difference	95% Confidence Intervals		*P*	% change
				Lower	Upper		
Heel	New Worn	318.8 (73.0) 323.0 (86.6)	−4.2	−20.3	11.9	0.61	1%
Midfoot	New Worn	154.2 (61.2) 157.3 (84.4)	−3.1	−19.1	12.9	0.70	2%
1MTP	New Worn	318.5 (114.6) 316.8 (110.2)	1.7	−14.3	17.7	0.83	1%
2MTP	New Worn	316.2 (88.1) 315.5 (99.4)	0.6	−15.4	16.6	0.94	0%
345MTP	New Worn	266.6 (96.5) 275.6 (105.2)	−9.0	− 25.0	6.9	0.27	3%
Hallux	New Worn	284.3 (124.3) 277.4 (137.3)	6.9	− 9.0	22.9	0.39	2%
Lesser toes	New Worn	188.1 (83.8) 192.2 (107.4)	−4.1	−20.1	11.8	0.61	2%

**Table 4:** Pressure time integrals (kPa*s)

Parameter	Condition	Mean (SD)	Difference	95% Confidence Intervals		*P*	% change
				Lower	Upper		
Heel	New Worn	43.8 (10.5) 44.1 (11.4)	−0.3	−1.5	0.9	0.60	1%
Midfoot	New Worn	32.5 (11.6) 32.7 (12.5)	−0.2	−1.4	1	0.75	1%
1MTP	New Worn	50.9 (20.1) 46.7 (15.7)	4.2	3.0	5.3	**< 0.001**	9%
2MTP	New Worn	48.9 (15.3) 46.3 (13.9)	2.6	1.4	3.7	**< 0.001**	6%
345MTP	New Worn	45.9 (16.4) 45.1 (16.3)	0.8	−0.4	2.0	0.18	2%
Hallux	New Worn	31.6 (13.5) 29.5 (12.5)	2.2	1.0	3.3	**0.003**	7%
Lesser toes	New Worn	25.2 (10.9) 24.0 (12.7)	1.2	−0.02	2.3	0.05	5%

## Discussion

This study is the first to report on footwear characteristics and plantar pressures in people with gout over 6 months. Our key findings show reduced pressure time integrals were observed at 1MTP, 2MTP and the hallux in the worn footwear. We also observed a reduction in heel counter and midfoot stiffness, an increase in midsole hardness, together with signs of upper, midsole and outsole wear occurred following six-months of use.

No differences were observed in peak plantar pressures between the footwear conditions in people with gout. This may be due to the normal wear patterns and the amount of degradation in the structural properties of the footwear over 6 months were not large enough to have a significant impact on peak plantar pressures. Increases in peak plantar pressure have been reported in older adults when comparing footwear with hard midsoles to soft midsoles [15], however, the differences in hardness between the footwear conditions in this study was 5% or less. Previous work has reported that people with gout frequently wear shoes that are of poor-quality and over 12 months old [2]. The footwear used in this study was constructed of materials that may be more resistant to wear compared to poor-quality footwear. The small wear patterns observed may also reflect a wearing-in period where the footwear becomes comfortable over time. In our clinical trial, improvements in footwear comfort and fit were observed over a six-month period whilst wearing the footwear evaluated in this study [8]. This suggests that good-quality footwear made of materials that offer cushioning and support are important for people with gout.

Reduced pressure time integrals at the hallux, 1MTP and 2MTP were observed in the worn footwear condition, which may be due to an immediate adaption to the new footwear at the study visit. Previous work in gout comparing new footwear with cushioning and a dual density midsole to the participants own worn footwear, reported a similar 6% reduction in pressure time integrals at the hallux in the worn footwear [4]. As patient reported outcomes were not collected as part of these studies it is unclear as to the impact these changes have on clinical outcomes. The reductions in pressure time integrals at these regions are also consistent with patterns previously reported in people with gout compared to controls when wearing worn footwear [5]. This lends further support to people with gout having reduced pressure time integrals under the hallux when walking in their own, worn footwear, which may be a pain-avoidance mechanism [5] or an adopted strategy [6, 11] to prevent triggering a flare [16]. The changes in loading observed in the worn footwear condition suggest a return to these abnormal gait patterns following wear over time in people with gout. Reductions in plantar pressure with rocker-sole footwear compared to the participant’s own footwear have been reported in first metatarsophalangeal joint osteoarthritis as a mechanism to improve patient outcomes [17]. The current knowledge about plantar pressures and clinical efficacy in gout is unknown and we can only speculate based upon current findings. Future work could look to explore the relationship between plantar pressures and clinical outcomes in gout.

The number of hours that the footwear was worn by participants was less than what has been reported in previous gout studies [3, 8]. This may be due to several reasons. People with gout have reported that footwear use is limited by flares, the appearance of the shoe and the different requirements of workplace and social settings [18]. The study was also conducted over a year, with seasonal differences known to affect footwear selection those with gout [19]. No minimum wear time was also prescribed to participants which may also have influenced footwear use. The adherence rates for diary completion suggest that future work should consider other strategies such as sensors [20] to record wear time.

This study has limitations. The footwear used in the study was high cost due to the quality of the shoe with its dual density midsole, heel and forefoot cushioning and a rocker profile. Our findings may not be translatable to other types of footwear, such as non-athletic footwear, or lower cost shoes with different material properties. The potential changes to the structural properties of the footwear, in the upper, midsole and outsole over a longer-period of time are not known, nor their influence on footwear comfort. Whether the observed changes to pressure time integrals translate into patient-centred outcomes such as foot pain and footwear comfort in people with gout is unknown. The diaries used to record footwear use are self-reported, did not record specific activities undertaken whilst wearing the footwear and may be subject to bias. The footwear fitting and data collection were undertaken by the same researcher, with researchers and participants unable to be blinded to the footwear conditions tested; however, bias was reduced by using a standardised protocol for assessments.

## Conclusion

In conclusion, there were reductions in heel and forefoot height, increases in midsole hardness and normal upper and outsole wear patterns following six-months of footwear use. These changes in the mechanical properties of the footwear may impact foot function, as observed by alterations in forefoot loading patterns between new and worn footwear.

## Data Availability

Data and material available for this study would require further approval upon request from the corresponding author.

## Abbreviations

BMI: Body mass index
1MTP: First metatarsal
345MTP: Lesser metatarsals
NSAID: Non-steroidal anti-inflammatory drug
2MTP: Second metatarsal
